# Unveiling mechanisms of change in digital interventions for depression: Study protocol for a systematic review and individual participant data meta-analysis

**DOI:** 10.3389/fpsyt.2022.899115

**Published:** 2022-10-03

**Authors:** Matthias Domhardt, Simon Grund, Axel Mayer, Rebekka Büscher, David D. Ebert, Lasse B. Sander, Eirini Karyotaki, Pim Cuijpers, Harald Baumeister

**Affiliations:** ^1^Department of Clinical Psychology and Psychotherapy, Ulm University, Ulm, Germany; ^2^Department of Quantitative Psychology, University of Hamburg, Hamburg, Germany; ^3^Department of Psychological Methods and Evaluation, Bielefeld University, Bielefeld, Germany; ^4^Department of Rehabilitation Psychology and Psychotherapy, University of Freiburg, Freiburg, Germany; ^5^Medical Psychology and Medical Sociology, Medical Faculty, University of Freiburg, Freiburg, Germany; ^6^Department of Psychology and Digital Mental Health Care, Technical University Munich, Munich, Germany; ^7^Department of Clinical, Neuro- and Developmental Psychology, Amsterdam Public Health Research Institute, Vrije Universiteit Amsterdam, Amsterdam, Netherlands

**Keywords:** depression, digital mental health, therapeutic process, mediator, psychotherapy

## Abstract

**Introduction:**

The efficacy and effectiveness of digital interventions for depression are both well-established. However, precise effect size estimates for mediators transmitting the effects of digital interventions are not available; and integrative insights on the specific mechanisms of change in internet- and mobile-based interventions (IMIs)—as related to key features like delivery type, accompanying support and theoretical foundation—are largely pending.

**Objective:**

We will conduct a systematic review and individual participant data meta-analysis (IPD-MA) evaluating the mediators associated with therapeutic change in various IMIs for depression in adults.

**Methods:**

We will use three electronic databases (i.e., Embase, Medline/PubMed, PsycINFO) as well as an already established database of IPD to identify relevant published and unpublished studies. We will include (1) randomized controlled trials that examine (2) mediators of (3) guided and unguided (4) IMIs with (5) various theoretical orientations for (6) adults with (7) clinically relevant symptoms of depression (8) compared to an active or passive control condition (9) with depression symptom severity as primary outcome. Study selection, data extraction, as well as quality and risk of bias (RoB) assessment will be done independently by two reviewers. Corresponding authors of eligible primary studies will be invited to share their IPD for this meta-analytic study. In a 1-stage IPD-MA, mediation analyses (e.g., on potential mediators like self-efficacy, emotion regulation or problem solving) will be performed using a multilevel structural equation modeling approach within a random-effects framework. Indirect effects will be estimated, with multiple imputation for missing data; the overall model fit will be evaluated and statistical heterogeneity will be assessed. Furthermore, we will investigate if indirect effects are moderated by different variables on participant- (e.g., age, sex/gender, symptom severity), study- (e.g., quality, studies evaluating the temporal ordering of changes in mediators and outcomes), and intervention-level (e.g., theoretical foundation, delivery type, guidance).

**Discussion:**

This systematic review and IPD-MA will generate comprehensive information on the differential strength of mediators and associated therapeutic processes in digital interventions for depression. The findings might contribute to the empirically-informed advancement of psychotherapeutic interventions, leading to more effective interventions and improved treatment outcomes in digital mental health. Besides, with our novel approach to mediation analyses with IPD-MA, we might also add to a methodological progression of evidence-synthesis in psychotherapy process research.

**Study registration with Open Science Framework (OSF):**

https://osf.io/md7pq/.

## Introduction

Depression is among the most common disorders across cultures worldwide (1), and is associated with substantial disease burden (2, 3). Although the evidence base for effective psychological and pharmacological treatments for depressive disorders is well-established (4–10), limited health care resources confine available treatment supplies; and several individual and structural access barriers, like stigma threat or high treatment costs, further restrict service utilization (11, 12). As a consequence, many patients receive no evidence-based treatment (13).

Digital psychotherapeutic interventions, such as internet- and mobile-based interventions (IMIs) might constitute one way in overcoming these undersupplies, given several assets when compared with “conventional” psychotherapies (i.e., empirically-supported psychotherapeutic interventions that are delivered face-to-face) such as possible anonymity or flexible utilization irrespective of space and time as well as their presumed potential to cost-effectively expand evidence-based treatment options (14, 15). The efficacy and effectiveness of IMIs for depression is established through several dozens of randomized controlled trials (RCTs) by now (16). Meta-analytical evidence of the few existing non-inferiority trials indicates that guided internet-based interventions achieve similar effect-sizes when compared to operationalizations of face-to-face psychotherapies of similar intensity and length at post-treatment (16–18). By contrast, self-guided internet-based interventions achieve typically smaller effect sizes than guided interventions on average (16–20), especially among patients with moderate to severe depression (21). Yet, completely unguided interventions might hold a bigger potential for up-scaling, as they can be entirely technology-delivered at relatively low costs (19). Mobile-based interventions (i.e., mental health apps) are particularly attractive when it comes to directly influence behavior change in critical moments in daily live (22, 23); still, they are often not scientifically informed (24–27) and evince smaller effect sizes than internet-based interventions on average (16, 28). Since the evidence on the efficacy and effectiveness of IMIs is well-established, the question of interest is moving to whereby, how and for whom these interventions work (29).

Research on the mechanisms of therapeutic change in IMIs is of high scientific and clinical relevance, as it possesses the potential to highlight avenues to further improve these interventions, eventually resulting in more specific and efficient interventions with higher treatment proficiency and larger effect sizes (30, 31). Knowledge about the mechanisms of change in psychotherapeutic interventions that is informative for the evidence-based advancement of interventions can be derived both from component and mediation studies. In component studies, psychotherapeutic interventions with multiple components (or modules) are experimentally compared with disentangled variations of the same intervention (32) in which either a component is left out (i.e., dismantling studies) or added to the full treatment package (i.e., additive design studies). These studies can lead to causal evidence on the specific effects of single intervention components (i.e., the active ingredients and effective therapeutic techniques and strategies whereby an intervention works) if they are conducted within a framework of a randomized controlled trial (33). However, knowledge derived from component studies does not fully reveal how a treatment works and which processes within patients actually lead to positive outcomes. Mediation studies can contribute to disclose these critical insights on change mechanisms and are often considered as an indispensable approach of psychotherapy process research. Mediation analyses can demonstrate statistically if a mediator variable transmits the effect of the treatment on the outcome (31). Thereby, a mediator might point to a possible mechanism of change (i.e., the processes within patients that are induced by intervention components and lead to change in the outcome), but may be also a proxy for one or more other variables actually representative for the true mechanism (31, 34). Therefore, several other criteria determine the capacity to draw causal conclusions in mediation analyses (31), with the establishment that the mediator variable changes before the outcome (i.e., temporal precedence) being of particular importance (35). Overall, it is a persistent problem of psychotherapy research that single component and mediation studies often lack the statistical power to reveal significant findings (29).

The results of individual component and mediation studies can be synthesized by systematic reviews and meta-analyses, and more recently by individual participant data meta-analyses (IPD-MAs). In contrast to conventional meta-analyses, which rely on aggregated data extracted from published studies, IPD-MAs seek to identify, collect, check, harmonize and analyse the data on individual participant level of eligible primary studies provided by corresponding authors (36). Hence, the sophisticated methodological approach of IPD-MA offers several advantages over conventional meta-analytic reviews (37), which might be especially relevant to psychotherapy research on change mechanisms. First, they possess substantially more statistical power as they combine IPD of single studies (38), resolving the lack of power in component studies (33) and mediation analyses in single studies and conventional meta-analyses (39). Second, they allow for the harmonization of variables (e.g., mediator and outcome instruments, measurement points) and standardization of analyses across studies (e.g., imputation strategies), facilitating investigations on multiple levels not possible in conventional meta-analyses (40). Third, IPD-MA go beyond analyses of aggregated data on study level and enable moderator analyses on the individual level that can generate important information for the personalization of psychotherapy (41). Fourth, data of published studies can be verified, missing data can be consistently accounted for at the individual level, and results of unpublished studies can be incorporated, reducing the problem of biased findings (38, 40). Given these assets, IPD-MA are considered to be the new gold standard in evidence synthesis (36).

The empirical findings on intervention components of internet-based cognitive behavioral therapy (iCBT) were recently meta-analytically evaluated by an IPD-MA using a network methodology approach (42). In their innovative study, Furukawa et al. (42) found strong evidence that the behavioral activation component (incremental mean difference (iMD) of PHQ-9 scores with 95% confidence intervals: −1.83; −2.90 to −0.80) and non-specific treatment effects components (iMD: −1.41; −2.52 to −0.30) were beneficial for the reduction of depression symptom severity; in contrast, there was initial evidence that the relaxation component had unfavorable effects (iMD: 1.20; 0.17 to 2.27) and could be considered as a detrimental component in iCBT for depression. The intervention component behavior therapy for insomnia was found to be helpful (iMD: −1.82; −3.92 to 0.26), yet based only on four component studies with weak evidence. Furthermore, there were indications that human encouragement in combination with automated encouragement decreases dropout from treatment [combined incremental odds ratio (iOR): 0.32; 0.13 to 0.93] and might be able to endorse therapy efficacy (combined iMD: −0.55; −1.75 to 0.65). Surprisingly, this IPD-MA (42) did not reveal statistical significant effects for therapeutic guidance (iMD: 0.01; −0.88 to 0.89), although it is otherwise well-established that guided iCBT is superior to unguided iCBT, especially among patients with higher baseline severity of depression (16, 21).

Yet, with this recent IPD-MA (42) the questions which mediators and actual therapeutic processes within patients lead to positive symptom change remain unanswered; since the completion of intervention components does not necessarily signify that patients have learned the respective skills and apply them in their daily lives. Furthermore, the IPD-MA on the components of iCBT could not yield differential findings on other evidence-based theoretical approaches for depression, such as problem-solving, psychodynamic or interpersonal therapy (43). Although we have already conducted a systematic review—without meta-analysis—on the mediators of digital interventions for depression before (44), we are not aware of any systematic study that has meta-analytically evaluated and quantified the individual strengths of mediators and mediator groups, neither with aggregated data nor with IPD so far. Thus, with this upcoming systematic review and IPD-MA on the mediators and mechanisms of change in IMIs for depression, we intend to close this gap and strive to accomplish the following research aims:

1), to systematically review studies on digital interventions for depression, in order to identify RCTs with published or unpublished individual participant data on potential mediators;
2), to pool individual participant data, in order to examine mediators and mechanisms of change in IMIs for depression;
3), to assess the magnitude and strength of mediators with identical or high conceptual overlap (i.e., ascertain precise effect size estimates for individual mediators and mediator groups);
4), to investigate if indirect effects are moderated by different variables on the participant, study, or intervention level (i.e., moderated mediation).

## Methods

### Registration and funding

This systematic review and IPD-MA is a priori registered with the Open Science Framework (https://osf.io/md7pq/). The study protocol is reported in accordance to the Preferred reporting items for systematic reviews and meta-analyses (PRISMA), as specified for protocols (45) and IPD-MA (36). The study is part of the project “*howIMIwork*,” funded by the German Federal Ministry of Education and Research (BMBF; grant identification: FKZ 01KG1802).

### Eligibility criteria

We will include studies if they meet the following inclusion criteria:

#### Participants

Adults (18 years or older) with clinically relevant depressive symptoms assessed with validated/standardized self-report or observer-rated instruments (i.e., depression score above the cut-off on the respective instrument). Alternatively, participants meet diagnostic criteria for a depressive disorder (ICD or DSM) based on diagnostic interview or clinician judgement.

#### Interventions

Digital interventions (i.e., internet-, mobile- or tablet-based interventions) with a psychotherapeutic focus for the acute treatment of depression will be included. Interventions have a theoretical foundation and might feature various operationalizations and types of accompanying (human and automated) support or might be pure self-help interventions. We did not set a minimum or maximum on the lengths of interventions. Interventions within a setting of blended therapy (i.e., mixed digital and face-to-face interventions) and group therapy will be excluded. Likewise, interventions that are based on teletherapy/telemedicine approaches (via telephone, voice over internet protocol, video-calls, videoconferencing or group chats) or interventions that merely rely on text messages will be excluded.

#### Comparators/control conditions

Both, active (e.g., other active treatment, care-as-usual, psychological placebo or attention control) and passive control conditions (e.g., waitlist control, no treatment) are eligible.

#### Primary outcomes

Depression symptom severity and mediators (both measured with validated instruments) at post-treatment or follow-up as assessed by self-report questionnaires (e.g., Beck Depression Inventory, Center for Epidemiological Studies Depression Scale or Patient Health Questionnaire) or standardized interviews (e.g., Hamilton Rating Scale for Depression or Montgomery-Åsberg Depression Rating Scale). Possible mediators (such as functional cognitions, self-efficacy or mindfulness skills) are to be measured with psychometric and dimensional instruments (e.g., Dysfunctional Attitudes Scale). A list of empirically-based mediators that are to be examined (see Table 1), is informed by two systematic reviews on mediators in digital interventions (44) and face-to-face psychotherapies for depression (39).

**Table 1:** List of potential mediators.

**Cognitive mediators**	**Behavioral mediators**	**Emotional and affective mediators**	**Skills as mediators**	**Other mediators**
Attention bias Attributional style Automatic thoughts Cognitive (de)fusion Cognitive distortions Dysfunctional attitudes and thinking Interpretation bias Memory specificity Metacognitive beliefs Negative thinking or thoughts Perceived control/Mastery Positive metacognitive beliefs about rumination/RNT Positive beliefs Positivity bias Psychological flexibility Repetitive negative thinking (RNT) Reappraisal Rumination Self-efficacy Worrying	Activation level Behavioral activation Self-help behavior	Emotion regulation Emotion regulation (cognitive reappraisal) Emotion regulation (suppression) Emotional self-awareness Positive and negative affect	CBT Skills Cognitive skills Coping Dispositional mindfulness Mindfulness Problem solving Problem solving skills (avoidance) Problem solving skills (impulsivity) Problem solving skills (negative or positive problem orientation) Problem solving skills (rational problem solving) Social skills	Acceptance Anxiety Attachment Ego-despair Ego-integrity Expectancy for improvements/Treatment expectancy Goal re-engagement Helplessness HIV related stigma Mental health literacy Perceived stress Perfectionism Relaxation Savoring (of pleasant events) Self-compassion Self-esteem Sense of autonomy Therapeutic alliance

#### Eligible secondary outcomes

Intervention adherence, study drop-out, adverse events, deterioration and quality of life (QoL).

#### Study design and publication type

RCTs that have evaluated mediator variables in a pre–post-design (i.e., with at least two measurement points) and/or have conducted one way of mediation analysis; studies have to be published in a peer-reviewed journal. Furthermore, we will also include efficacy and effectiveness trials that have unpublished data on mediators and are part of an already existing IPD pool (see below). No restrictions in regard to language will be upheld; although a title and an abstract in English language must be available, in order to enable initial screening for eligibility.

### Information sources, search strategy, and study selection process

This IPD-MA builds upon an existing IPD database established by Cuijpers and Karyotaki [e.g., (19, 21, 46)]. The IPD database will be updated and further complemented by newly identified studies through a systematic literature search conducted on February 25, 2022. The databases of Embase, Medline/PubMed, and PsycINFO are searched according to a predefined set of search strings for each database with no restriction on dates of coverage and language (see the Appendix for details on all search strings). All corresponding authors of included trials will be contacted and asked about other RCTs—published or unpublished—which might be relevant to this IPD-MA.

The selection of eligible primary studies will be conducted by two independent reviewers. If title and abstract contain sufficient information to determine exclusion, the article will be rejected. The full papers of all remaining articles will be retrieved and then reviewed by the two independent reviewers. In addition, all other potentially relevant articles identified by checking the reference lists or personal communications will be reviewed. A record of the reasons for rejection will be documented in a PRISMA flow chart. All eligible papers not published in English will be translated into English. If the two reviewers disagree about the inclusion of an article, a third reviewer will be asked to review the article. Disagreements will then be solved by discussion.

### Data collection process

First, we will check the existing IPD database if eligible studies are already part of the IP data pool. Second, the corresponding first or senior authors of eligible RCTs identified additionally by the systematic literature searches will be contacted by email and asked for the data necessary for the present IPD-MA. If there is no reply, reminders will be sent 2 and 4 weeks after the first contact by email to both first and senior authors. If there is no response after 1 month or the authors cannot provide the IPD, the trial will be excluded as not available.

### Data items

Two independent reviewers will extract a range of IPD, aggregated data and other information from eligible studies, both from the IPD datasets provided and the corresponding published papers. Data on the individual level comprise information on sociodemographic, clinical, and intervention characteristics, including information regarding randomized group, scores of depressive symptom severity (at baseline, post-treatment and follow-up), mediator variable scores on all measurement points, treatment adherence information, age, sex/gender, educational level, employment status, relationship status, and comorbid symptoms at baseline. Information will be requested for all patients randomized (i.e., all participants initially assigned to a treatment group), including those that were excluded from the investigators' own analyses, in order to perform Intention-to-treat analysis (ITT). Furthermore, we will request information on IMI-specific features such as usage of automated encouragement, human support (i.e., guidance), delivery mode (internet-, mobile- or tablet-based), or theoretical orientation/therapeutic approach of the intervention.

Study-level information will include references, year of publication, country, recruitment setting, intervention and control/comparison groups, number of participants per group and percentage of female participants, mean age, intervention adherence, study drop-out, number of modules and duration in weeks, follow-up, measures of symptom severity, potential mediators, statistical method for mediation analyses, clinical outcomes, and unpublished or additional material. This study level information will be also extracted from published studies to check IPD-integrity.

Data will be harmonized across studies in case of different definitions and operationalizations of variables. First, a harmonized version of the raw IPD dataset will be prepared for each study separately. After this, we will merge all harmonized IPD datasets in a comprehensive and integrated IPD-MA dataset that will be used for the mediation analyses. All randomized participants will be included into the merged IPD dataset, in order to secure ITT-analyses.

### IPD integrity

All data will be thoroughly checked for validity, completeness and consistency by two independent reviewers. We will compare descriptive analyses of IPD (means, standard deviations and percentages of all (continuous) clinical variables and demographics at baseline, during and post-treatment) per study with the published results and information of primary studies in order to check IPD integrity. Inconsistencies and unusual patterns will be clarified with trialists. Final trial quality assurance analyses will be sent to trialists for verification.

### Risk of bias assessment in individual studies

Two independent reviewers will assess the risk of bias of all included studies using the Cochrane‘s revised Risk of bias tool (RoB 2) for randomized trials (47). The RoB 2 includes the following five domains: (a) bias arising from the randomization process; (b) bias due to deviations from intended interventions; (c) bias due to missing outcome data; (d) bias in measurement of outcome; and (e) bias in selection of the reported outcome. Because masking of participants (and therapists) is not possible in psychotherapy research (48, 49), self-report measurements of outcome would ultimately lead to a high risk of bias; thus, the fourth criterion of the RoB 2 will be not rated in this IPD-MA given the specifics of psychotherapy research. Disagreements in the RoB 2 assessment will be solved through discussion or by consultation of a third reviewer.

### Quality criteria of process research

In order to evaluate the appropriateness of research designs of included studies to detect mechanisms of change and to approach causality, a rating system based on the criteria proposed by Kazdin (31) and adapted to psychotherapy research for common mental disorders by Lemmens et al. (39), Domhardt et al. (34, 35, 44, 50), and Steubl et al. (51, 52) will be applied. Studies will be rated in regard to the following nine criteria as either fulfilled or not fulfilled independently by two reviewers: (a) utilization of an appropriate RCT design, (b) inclusion of a control group, (c) report of a theoretical foundation for mediators, (d) minimum sample size of 40 participants per group, (e) examination of multiple mediators within one study, (f) assessment of temporality (three or more assessments of the mediator variables and outcomes), (g) experimental manipulation of the mediator, (h) significant statistical association between treatment, mediator and outcome (*p* < 0.05) and (i) consideration and control of confounding variables in a methodologically appropriate manner (35, 53). Studies will be divided into high vs. low-quality studies; and studies that have evaluated the crucial criterion (f) of the temporal precedence of changes in the mediator before the outcome or those that did not.

### Specification of outcomes and effect measures

Depression symptom severity as assessed with standardized and continuous observer-rated or self-report instruments at post-treatment will be the primary outcome. All validated depression outcome instruments (such as the Beck Depression Inventory-II, Hamilton Rating Scale for Depression or Patient Health Questionnaire-9) are eligible. If both observer-rated and self-report measures are used in one study, preference will be given to the measure reported by the majority of the included studies in order to increase comparability. In case the types of outcome instruments vary between studies, these measures will be transformed into converted/standardized scores using the common metric approach described by Wahl et al. (54), complemented with alternative approaches described by Furukawa et al. (55) and Choi et al. (56) if necessary. If this procedure is not possible, scale scores will be standardized and transformed into z-scores to generate a common metric for depression symptom severity within and across studies.

Next to depression symptom severity, a range of mediators (such as perceived control, mastery, psychological flexibility, repetitive negative thinking or problem solving) are considered to be additional primary outcomes and of main interest to this IPD-MA. Mediators are to be measured with validated continuous self-reports or observer-rated instruments. If both self-report and observer-rated measures are used in one study, preference will be given to the most frequently used measures across the different studies in order to facilitate synthetization and harmonization of mediators between studies. In case a study has evaluated more than one mediator, all mediators are included into the IPD-MA. A list of all potential mediators (grouped into cognitive, behavioral, emotional/affective, skills and other categories) that are to be evaluated in this IPD-MA can be found in Table 1.

A range of secondary outcomes are to be analyzed in this study as well. Intervention adherence (operationalized on the individual-level (a) as the percentage of main intervention units (i.e., components or modules) completed; and on study-level, both (b) as the percentage of participants that completed the whole treatment, as well as (c) the dropout rates at post-treatment), adverse events (e.g., crisis intervention because of suicidality), symptom deterioration and QoL as reported in the primary studies and IPD.

### Synthesis methods and mediation analyses

Since the seminal paper of Baron and Kenny (57), research on mediational analysis has evolved, both in least squares regression approaches and in the framework of structural equation modeling (SEM). These two analytic methods are most often applied in the context of mediational analysis in clinical psychology and psychotherapy research (58, 59), though the SEM framework offers several advantages in comparison to standard regression approaches for mediation analysis: SEM (1) allows for simultaneous consideration of multiple independent variables, mediators and outcomes, (2) provides model fit information, (3) can be extended to latent variables as well as longitudinal data with temporal ordering, (4) can be applied in a multilevel context, and (5) enables an expression of a functional relationship via a conceptual model. Thus, we plan to analyse mediation with current SEM methods in our IPD-MA, using a 1-stage approach [1-stage IPD-MA (60)] with random-effects.

For separate mediation analyses, we will combine all individual participant data from all studies, taking into account the multilevel structure of the data [e.g., (61, 62)], and implement novel multilevel meta-analytic SEM techniques. In line with prior conventional meta-analyses (35, 63), we do not intend to conduct meta-analyses if less than three effect sizes for one analysis/comparison are available. For each analysis, mediators will be grouped and harmonized into the following categories: (1) mediators with identical/high conceptual overlap, either assessed with the same instrument, or with differing instruments; and (2) mediators with sufficient conceptual overlap evaluated with different measurements. First, the scale scores of mediators in these groups will be standardized (transformed into z-scores) to create a common metric for the respective mediator with identical/high or sufficient conceptual overlap to be synthesized for the mediation analyses in this IPD-MA. Missing values will be handled with multiple imputation or Bayesian estimation techniques for multilevel data [e.g., (64–68)]. In a second step, we will then analyse mediation using multilevel SEM that simultaneously models relationships between intervention, mediators and outcomes in a random-effects framework for each mediator (group) separately. In this context, we will use Maximum Likelihood (ML) or Bayesian estimation procedures and include random effects for the mediator and outcome variables as well as for the direct and indirect effects. The strength and significance of indirect effects in these mediational models (for each mediator or mediator groups with identical or high conceptual overlap) will be estimated by the delta-method and bootstrap approaches (69, 70) in combination with multiple imputation (71). In these multilevel mediation models we aim to model each path with random effects wherever appropriate. We will further provide percentile confidence intervals (CIs) and bootstrapped standard errors. Sensitivity analysis for causal mediation analysis will be undertaken as proposed by Imai et al. (72). Third, the model fit, which represents how an SEM fits with the data, will be assessed using common indices adjusted to the current IPD-MA.

Statistical heterogeneity will be assessed with the *I*^2^ statistic and the random effects variance τ (73). Possible publication bias will be examined with Egger's test of funnel plot asymmetry (74) and visual inspection of funnel plots.

Data analyses will be performed with the statistical programs R (75) and Mplus (76). Inter-rater reliabilities (using Cohen's Kappa) will be computed for risk of bias assessment and the criteria for process research using the R package *irr* [version 0.84.1 (77)].

In case that individual participant data will not be available for all relevant eligible trials or a 1-stage approach is unfeasible with the available data, we intend to resort to meta-analysis methods for combining individual participant data with aggregated data (2-stage IPD-MA). All necessary modifications of the planned procedures detailed in this study protocol will be transparently and comprehensively outlined in an amendment to this IPD-MA.

### Exploration of variation in effects

We intend to conduct several exploratory moderator and subgroup analyses within this IPD-MA. If feasible the following potential moderators of indirect effects (i.e., moderated mediation) on participant-, study-, and intervention-level are to be investigated as suggested by prior research (16, 19, 21, 41, 44, 62, 78, 79): (a) participant characteristics (e.g., age, sex/gender), (b) baseline symptom severity, (c) comorbidity, (d) low vs. high-quality studies in regard to quality criteria for process research, (e) studies evaluating the temporal ordering of changes in mediators and outcomes vs. studies that did not, (f) type of psychotherapeutic approach/theoretical orientation of the intervention, (g) delivery mode, and (h) type of guidance (e.g., fully self-guided/unguided, automated encouragement, human support). Furthermore, (i) other potential moderators, as analysable with the final sample of eligible primary studies, might be exploratory examined.

### Risk of bias across studies

We will assess the potential risk of bias across studies, relating to the accumulated body of evidence within this IPD-MA as well (36, 80). Furthermore, we intend to rate the RoB-2 based also on the IPD conveyed by the trialists as described by Büscher et al. (81). This might lead to lessened RoB-2-ratings compared to the standard RoB-2-assessment (based on the information provided in the publications) in some instances, since we might be able to amend existing risks of bias of primary studies by resorting and analyzing all IPD. For example, in the situation that the RoB-2 is rated high due to selective outcome reporting in the publication, we can principally include all outcomes and mediators in our analyses based on the IPD provided, resulting in a low RoB-2 rating for the accumulated evidence in the IPD-MA (81).

## Ethics and dissemination

Although this systematic review and IPD-MA does not require institutional review board (IRB) approval as it resorts to already collected data from studies with ethical approval only, the project was approved by the ethics committee of the German Psychological Society (DGPs; BaumeisterHarald2020-08-11VADM). Before data transfer, corresponding investigators are encouraged to anonymise all IPD so that it is impossible to identify individual trial participants. Data transfer will follow a protocol (e.g., password-protected encryption; way of data transfer), but might vary depending on the data security requirements from the participating institutions. The IPD datasets will be recorded on secure institutional servers of the Vrije Universiteit Amsterdam. Data provided for the IPD-MA will remain the property of researchers providing the data. We will deploy an IPD-policy-form with information in regard to data-ownership, -confidentiality, -access and -use as well as rules for authorship.

First and senior authors of eligible primary studies will be invited for co-authorship for all publications on this IPD-MA based on their data, in line with the standards of good scientific practice (82). We will submit a manuscript on the results of this study to an international peer-reviewed journal dedicated to psychotherapy research, clinical psychology/psychiatry and digital mental health, in accordance to the PRISMA statements (36, 83). Additionally, we will present the results on international conferences with a focus on psychotherapy and internet intervention research in order to reach scientists, stakeholders, policy makers and clinicians alike.

## Discussion

This systematic review and IPD-MA will contribute to unveil the mechanisms of change in digital interventions, and generate precise effect size estimates for mediators in IMIs for depression. Therewith, the proposed IPD-MA has the potential to provide comprehensive and integrative information for the evidence-based development of interventions and the efficient implementation of IMIs in routine care, eventually enabling more effective interventions and improved treatment outcomes in digital mental health. Furthermore, it might also add to a methodological progression of evidence-synthesis in (psychotherapy) process research in general, since to the knowledge of the authors, this study is the first systematic review that conducts mediation analyses within a framework of IPD-MA.

The pre-planned subgroup analyses (i.e., moderated mediation) on a range of key variables on patient- (such as baseline symptom severity, age, sex/gender) and intervention-level (like delivery type, accompanying support/guidance, theoretical foundation) might yield important findings that are informative for precision digital mental health (29), facilitating differential pre-treatment decisions on which digital intervention and inherent therapeutic mechanisms work best for individual patients. Furthermore, the expected findings on secondary outcomes might also shed light on central mechanisms associated with intervention adherence, drop out, deterioration and adverse events, illustrating ways to further address these central issues in digital mental health. The novel possibilities of a digitalised psychotherapy process research with IMIs—for example in terms of a higher standardization of interventions, experimental manipulation of mediators with reduced complexity as well as ecological momentary assessments and passive smart-sensing studies on real-life mechanisms—might enable findings that were not conceivable for “conventional” psychotherapy research conducted face-to-face (29). Moreover, the intended comparison of the mediators in conventional face-to-face psychotherapies with the results of this IPD-MA on digital interventions will identify shared and distinct evidence-based mechanisms of change in psychotherapeutic interventions for depression, highlighting potential avenues to further increase the proficiency of each treatment format and how to best integrate both approaches in future blended mental health care (84).

Several strengths of this study are associated with the IPD-MA-approach, including the standardization of variables across studies, the treatment of missing data at the individual level, the verification of IPD against the information in published papers, the novel approach of mediation analyses with IPD, and the corresponding increase in statistical power to ascertain effect size estimates more precisely. Nonetheless, this IPD-MA might be also confronted with significant challenges and possible limitations. First, although the current study can build upon an already established IPD database, it might be that not all relevant primary studies are identified with the systematic literature searches; and we might face difficulties to obtain all additional and necessary IPD from all eligible primary studies, for example due to legal constraints or the unavailability of data. However, a comparison of the results of the 1-stage IPD-MA with the results of an optional 2-stage IPD-MA comprising all relevant studies might lessen this possible limitation. Second, the harmonization and standardization of mediation studies might be especially challenging, given the heterogeneity of this research field (35, 44, 50, 51, 85) and the large number of data points and variables that have to be synthesized. Third, only a minority of mediation studies have implemented a longitudinal design with at least three assessments points of mediators and outcomes (44), principally enabling the examination of the temporal ordering and patterns of changes in mediators and outcomes; in consequence, the scope of causal conclusions might be constrained—beyond the inherent limitations of statistical mediation itself (86). Fourth, the meta-analytic mediation analyses might be restricted by a limited amount of identical mediators and mediator groups with sufficient conceptual overlap that were examined in primary studies. Thus, not all mediator variables of interest might be evaluated in this IPD-MA. Fifth, some biases that are not completely solvable by the IPD-MA-approach (e.g., bias arising from the randomization process or bias due to deviations from intended interventions) have to be considered and accounted for.

In conclusion, the IPD-MA-project “*howIMIwork*” will contribute to augmented and integrative insights on the mediators and mechanisms of change in IMIs for depression and thus might have important implications for the advancement of digital mental health and psychotherapy process research. Future patients might benefit from the results of this proposed IPD-MA in that the findings will guide the evidence-based advancement of digital interventions for depression, which can lead to more efficient and safer interventions, with improved efficacy and higher adherence rates. This is especially important, as IMIs have got the potential to cost-effectively scale up treatment options for a substantial number of patients worldwide, thereby contributing to a significant reduction in the disease burden and alleviation of personal suffering associated with depression.

## Author contributions

MD, SG, AM, EK, PC, and HB developed the design of the study. MD wrote the first draft of the manuscript. All authors have contributed to the further writing and have approved the final manuscript.

## Funding

The project Mechanisms of change in internet- and mobile-based interventions for depression: A systematic review and meta-analysis of individual participant data - howIMIwork was funded by the German Federal Ministry of Education and Research (BMBF; Grant Identification FKZ 01KG1802). The funding source BMBF had no role in the study design, collection, analysis or interpretation of the data, writing the manuscript, or the decision to submit this paper for publication.

## Conflict of interest

MD reports to have received fees for lectures and workshops for different psychotherapy training institutes. LS reports to have received personal fees from psychotherapy training institutes, health insurance companies, and clinic providers in the context of e-mental-health outside the submitted work. DE reports to have received consultancy fees or served in the scientific advisory board from several companies such as Novartis, Sanofi, Lantern, Schön Kliniken, Minddistrict, and German health insurance companies BARMER, Techniker Krankenkasse. DE is stakeholder of the Institute for Health Trainings Online GET.ON, which aims to implement scientific findings related to digital health interventions into routine care. HB reports to have received consultancy fees, fees for lectures or workshops from chambers of psychotherapists and training institutes for psychotherapists and license fees for an Internet-intervention. The remaining authors declare that the research was conducted in the absence of any commercial or financial relationships that could be construed as a potential conflict of interest. The reviewer KM declared a past collaboration with the authors DE and EK to the handling editor.

## Publisher's note

All claims expressed in this article are solely those of the authors and do not necessarily represent those of their affiliated organizations, or those of the publisher, the editors and the reviewers. Any product that may be evaluated in this article, or claim that may be made by its manufacturer, is not guaranteed or endorsed by the publisher.
