# Supplementary material for: Unveiling mechanisms of change in digital interventions for depression: Study protocol for a systematic review and individual participant data meta-analysis
Source: Front Psychiatry. 2022 Oct 3;13:899115. doi: 10.3389/fpsyt.2022.899115 (PMC9574035; doi:10.3389/fpsyt.2022.899115)
Supplement: Supplementary file 1 [file Table_1.DOCX]

**Appendix**

Search strings for PubMed:

**# Depression**

"Depression"[Mesh] OR "Depressive Disorder"[Mesh] OR “depress*”[tiab] OR “dysthymi*”[tiab] OR “mood disorder*”[tiab] OR “affective disorder*”[tiab] OR “dysphoric disorder*”[tiab]

**# Psychological interventions**

“Psychotherapy”[Mesh] OR “Counseling”[Mesh] OR psychotherap*[Tiab] OR cbt[Tiab] OR counselling[Tiab] OR counseling[Tiab] OR “Eye Movement Desensitization Reprocessing”[tiab] OR “Eye Movement Desensitization and Reprocessing”[tiab] OR “Eye Movement Desensitisation Reprocessing”[tiab] OR “Eye Movement Desensitisation and Reprocessing”[tiab] OR EMDR[tiab] OR “Bibliotherap*”[tiab] OR mindfulness[Tiab] OR “Autogenic Training”[tiab] OR Logotherap*[tiab] OR "cognitive restructuring"[Tiab] OR "self-control training*"[Tiab] OR "assertiveness training"[Tiab] OR ((“therapy”[SubHeading] OR therap*[Tiab] OR “Therapeutics”[Mesh] OR treatment*[Tiab] OR intervention*[tiab]) AND (“brief psychodynamic”[Tiab] OR “short psychodynamic”[tiab] OR "problem-solving"[Tiab] OR "compassion-focused"[Tiab] OR "compassion-focussed"[Tiab] OR “compassion-based”[tiab] OR constructivist*[Tiab] OR metacognitive[tiab] OR “meta-cognitive”[Tiab] OR "solution-focused"[Tiab] OR "solution-focussed"[Tiab] OR "self-control”[Tiab] OR psychosocial[tiab] OR “peer support“[tiab] OR “task-shifted”[tiab] OR Relaxation[tiab] OR “dialectical behavior”[tiab] OR “emotion-focused”[tiab] OR narrative[tiab] OR “person-centred”[tiab] OR “person-centered”[tiab] OR “Narrative”[tiab] OR “meaning-centered”[tiab] OR “humanistic”[tiab] OR “client-centered”[tiab] OR “meaning-centred”[tiab] OR “client-centred”[tiab] OR “Rogerian”[tiab] OR “Nondirective”[tiab] OR “Non-directive”[tiab] OR “Supportive”[tiab] OR “Life review”[tiab] OR "acceptance and commitment"[Tiab] OR (“schema”[tiab] AND brief[tiab]) OR (“gestalt”[tiab] AND brief[tiab]))) OR "behavior therap*"[Tiab] OR "behaviors therap*"[Tiab] OR "behavioral therap*"[Tiab] OR "behaviour therap*"[Tiab] OR "behaviours therap*"[Tiab] OR "behavioural therap*"[Tiab] OR "cognition therap*"[Tiab] OR “cognitive therap*”[tiab] OR "behavior treatment*"[Tiab] OR "behaviors treatment*"[Tiab] OR "behavioral treatment*"[Tiab] OR "behaviour treatment*"[Tiab] OR "behaviours treatment*"[Tiab] OR "behavioural treatment*"[Tiab] OR "cognition treatment*"[Tiab] OR “cognitive treatment*”[tiab] OR "behavior intervention*"[Tiab] OR "behaviors intervention*"[Tiab] OR "behavioral intervention*"[Tiab] OR "behaviour intervention*"[Tiab] OR "behaviours intervention*"[Tiab] OR "behavioural intervention*"[Tiab] OR "cognition intervention*"[Tiab] OR “cognitive intervention*”[tiab] OR "behavior activation*"[Tiab] OR "behaviors activation*"[Tiab] OR "behavioral activation*"[Tiab] OR "behaviour activation*"[Tiab] OR "behaviours activation*"[Tiab] OR "behavioural activation*"[Tiab] OR exposure[tiab]

**# Internet-based**

"Telemedicine"[Mesh] OR "Mobile Applications"[Mesh] OR "Social Media"[Mesh] OR "Therapy, Computer-Assisted"[Mesh:NoExp] OR "Drug Therapy, Computer-Assisted"[Mesh:NoExp] OR "Telecommunications"[Mesh:NoExp] OR "Electronic Mail"[Mesh] OR "Videoconferencing"[Mesh] OR "Cell Phone"[Mesh] OR "Distance Counseling"[Mesh] OR “Wearable Electronic Devices”[Mesh] OR telehealth[tiab] OR “tele-health”[tiab] OR telepsychology[tiab] OR “tele-psychology”[tiab] OR telepsychiatry[tiab] OR “tele-psychiatry”[tiab] OR “tele-therap*”[tiab] OR teletherap*[tiab] OR “tele-medicine”[tiab] OR telemedicine[tiab] OR telecare[tiab] OR “tele-care”[tiab] OR telecommunicat*[tiab] OR “tele-communicat*”[tiab] OR teleconference*[tiab] OR “tele-conferenc*”[tiab] OR videoconferenc*[tiab] OR “video-conferenc*”[tiab] OR computer*[tiab] OR electronic*[tiab] OR digital*[tiab] OR ehealth[tiab] OR “e-health”[tiab] OR “e-treat*”[tiab] OR “e-therap*”[tiab] OR mhealth[tiab] OR “m-health”[tiab] OR “internet-based*”[tiab] OR “internet treat*”[tiab] OR “internet intervention*”[tiab] OR “internet counsel*”[tiab] OR “distance counsel*”[tiab] OR “web-based*”[tiab] OR cybercounsel*[tiab] OR “cyber-counsel*”[tiab] OR “online treat*”[tiab] OR “online therap*”[tiab] OR “online intervention*”[tiab] OR “online prevention*”[tiab] OR “online counsel*”[tiab] OR “text-messag*”[tiab] OR textmessag*[tiab] OR SMS[tiab] OR texting*[tiab] OR “short message service*”[tiab] OR mobile*[tiab] OR smartphone*[tiab] OR “cell-phone*”[tiab] OR cellphone*[tiab] OR “cellular phone*”[tiab] OR blended*[tiab] OR “software app*”[tiab] OR “handheld device*”[tiab] OR “hand held device*”[tiab] OR iPad*[tiab] OR iPhone*[tiab] OR email*[tiab] OR “e-mail*”[tiab] OR sensor*[tiab] OR wearable*[tiab] OR “social media*”[tiab] OR “social network*”[tiab] OR “e-counsel*”[tiab] OR ecounsel*[tiab] OR palmtop*[tiab] OR telephone*[tiab] OR WhatsApp[tiab] OR Twitter[tiab] OR Facebook[tiab] OR Instagram[tiab] OR forum[tiab] OR chat*[tiab] OR “virtual reality*”[tiab] OR avatar*[tiab] OR “Conversational agent*”[tiab] OR “virtual coach”[tiab] OR “virtual agent*”[tiab] OR “embodied agent*”[tiab] OR “relational agent*”[tiab] OR “interactive agent*”[tiab] OR “virtual character*”[tiab] OR “virtual human*”[tiab] OR “virtual assistant*”[tiab] OR VR[tiab] OR “serious game*”[tiab] OR “serious gaming”[tiab] OR gamification[tiab]

**# Trials/ SR/ Meta-analysis**

"Meta-Analysis" [Publication Type] OR "Meta-Analysis as Topic"[Mesh] OR metaanaly*[tiab] OR meta-analy*[tiab] or metanaly*[tiab] OR "Systematic Review" [Publication Type] OR systematic[sb] OR meta-analysis[Filter] OR systematicreview[Filter] OR "Cochrane Database Syst Rev"[Journal] or prisma[tiab] OR “preferred reporting items”[tiab] OR prospero[tiab] OR ((systemati*[ti] OR umbrella[ti] OR “structured literature”[ti]) AND (review[ti] OR overview[ti])) OR “systematic review”[tiab] OR “umbrella review”[tiab] OR “structured literature review”[tiab] OR “systematic qualitative review”[tiab] OR “systematic quantitative review”[tiab] OR “systematic search and review”[tiab] OR “systematized review”[tiab] OR “systematised review”[tiab] OR “systemic review”[tiab] OR “systematic literature review”[tiab] OR “systematic integrative literature review”[tiab] OR “systematically review”[tiab] OR “scoping literature review”[tiab] OR “scoping review”[tiab] OR “systematic critical review”[tiab] OR “systematic integrative review”[tiab] OR “systematic evidence review”[tiab] OR “systematic integrative literature review”[tiab] OR “systematic mixed studies review”[tiab] OR “systematized literature review”[tiab] OR “systematic overview”[tiab] OR “Systematic narrative review”[tiab] OR “narrative review”[tiab] OR metasynthes*[tiab] OR meta-synthes*[tiab] OR “Randomized Controlled Trial”[pt] OR “Randomized Controlled Trials as Topic”[Mesh] OR “Random allocation” [Mesh] OR “Double-blind method”[Mesh] OR “Single-blind method”[Mesh] OR random[tiab] OR randomly[tiab] OR randomised[tiab] OR randomized[tiab] OR randomising[tiab] OR randomizing [tiab] OR ((singl*[tiab] OR doubl*[tiab] OR trebl*[tiab] OR tripl*[tiab]) AND (mask*[tiab] OR blind*[tiab] OR dumm*[tiab])) OR RCT[tiab] OR "Clinical Trials as Topic"[Mesh] OR “Clinical Trial”[pt] OR “clinical trial*”[tiab] OR “Controlled Clinical Trial”[pt] OR “controlled trial*”[tiab]

**# Publication type**

NOT ("Comment" [Publication Type] OR "Letter" [Publication Type] OR "Editorial" [Publication Type] OR (("Animals"[Mesh] OR "Models, Animal"[Mesh]) NOT "Humans"[Mesh]))

**# Timeframe**

After 2000

Search strings for Embase:

**# Depression**

'depression'/exp OR ‘depress*’:ab,ti,kw OR ‘dysthymi*’:ab,ti,kw OR ‘mood disorder*’:ab,ti,kw OR ‘affective disorder*’:ab,ti,kw OR ‘dysphoric disorder*’:ab,ti,kw

**# Psychotherapy**

'psychotherapy'/exp OR 'counseling'/exp OR psychotherap*:ab,ti,kw OR cbt:ab,ti,kw OR counselling:ab,ti,kw OR counseling:ab,ti,kw OR ‘Eye Movement Desensitization Reprocessing’:ab,ti,kw OR ‘Eye Movement Desensitization and Reprocessing’:ab,ti,kw OR ‘Eye Movement Desensitisation Reprocessing’:ab,ti,kw OR ‘Eye Movement Desensitisation and Reprocessing’:ab,ti,kw OR EMDR:ab,ti,kw OR ‘Bibliotherap*’:ab,ti,kw OR mindfulness:ab,ti,kw OR ‘Autogenic Training’:ab,ti,kw OR Logotherap*:ab,ti,kw OR ‘cognitive restructuring’:ab,ti,kw OR ‘self-control training*’:ab,ti,kw OR ‘assertiveness training’:ab,ti,kw OR (('therapy'/lnk OR 'therapy'/exp OR therap*:ab,ti,kw OR treatment*:ab,ti,kw OR intervention*:ab,ti,kw) AND (‘brief psychodynamic’:ab,ti,kw OR ‘short psychodynamic’:ab,ti,kw OR ‘problem-solving’:ab,ti,kw OR ‘compassion-focused’:ab,ti,kw OR ‘compassion-focussed’:ab,ti,kw OR ‘compassion-based’:ab,ti,kw OR constructivist*:ab,ti,kw OR metacognitive:ab,ti,kw OR ‘meta-cognitive’:ab,ti,kw OR ‘solution-focused’:ab,ti,kw OR ‘solution-focussed’:ab,ti,kw OR ‘self-control’:ab,ti,kw OR psychosocial:ab,ti,kw OR ‘peer support’:ab,ti,kw OR ‘task-shifted’:ab,ti,kw OR Relaxation:ab,ti,kw OR ‘dialectical behavior’:ab,ti,kw OR ‘emotion-focused’:ab,ti,kw OR narrative:ab,ti,kw OR ‘person-centred’:ab,ti,kw OR ‘person-centered’:ab,ti,kw OR ‘Narrative’:ab,ti,kw OR ‘meaning-centered’:ab,ti,kw OR ‘humanistic’:ab,ti,kw OR ‘client-centered’:ab,ti,kw OR ‘meaning-centred’:ab,ti,kw OR ‘client-centred’:ab,ti,kw OR ‘Rogerian’:ab,ti,kw OR ‘Nondirective’:ab,ti,kw OR ‘Non-directive’:ab,ti,kw OR ‘Supportive’:ab,ti,kw OR ‘Life review’:ab,ti,kw OR ‘acceptance and commitment’:ab,ti,kw OR (‘schema’:ab,ti,kw AND brief:ab,ti,kw) OR (‘gestalt’:ab,ti,kw AND brief:ab,ti,kw))) OR ‘behavior therap*’:ab,ti,kw OR ‘behaviors therap*’:ab,ti,kw OR ‘behavioral therap*’:ab,ti,kw OR ‘behaviour therap*’:ab,ti,kw OR ‘behaviours therap*’:ab,ti,kw OR ‘behavioural therap*’:ab,ti,kw OR ‘cognition therap*’:ab,ti,kw OR ‘cognitive therap*’:ab,ti,kw OR ‘behavior treatment*’:ab,ti,kw OR ‘behaviors treatment*’:ab,ti,kw OR ‘behavioral treatment*’:ab,ti,kw OR ‘behaviour treatment*’:ab,ti,kw OR ‘behaviours treatment*’:ab,ti,kw OR ‘behavioural treatment*’:ab,ti,kw OR ‘cognition treatment*’:ab,ti,kw OR ‘cognitive treatment*’:ab,ti,kw OR ‘behavior intervention*’:ab,ti,kw OR ‘behaviors intervention*’:ab,ti,kw OR ‘behavioral intervention*’:ab,ti,kw OR ‘behaviour intervention*’:ab,ti,kw OR ‘behaviours intervention*’:ab,ti,kw OR ‘behavioural intervention*’:ab,ti,kw OR ‘cognition intervention*’:ab,ti,kw OR ‘cognitive intervention*’:ab,ti,kw OR ‘behavior activation*’:ab,ti,kw OR ‘behaviors activation*’:ab,ti,kw OR ‘behavioral activation*’:ab,ti,kw OR ‘behaviour activation*’:ab,ti,kw OR ‘behaviours activation*’:ab,ti,kw OR ‘behavioural activation*’:ab,ti,kw OR exposure:ab,ti,kw

**# Internet-based**

'telehealth'/exp OR 'mobile application'/exp OR 'social media'/exp OR 'computer assisted therapy'/de OR 'computer assisted drug therapy'/exp OR 'e-mail'/exp OR 'videoconferencing'/exp OR 'mobile phone'/exp OR 'wearable computer'/exp OR telehealth:ab,ti,kw OR ‘tele health’:ab,ti,kw OR telepsychology:ab,ti,kw OR ‘tele psychology’:ab,ti,kw OR telepsychiatry:ab,ti,kw OR ‘tele psychiatry’:ab,ti,kw OR ‘tele therap*’:ab,ti,kw OR teletherap*:ab,ti,kw OR ‘tele medicine’:ab,ti,kw OR telemedicine:ab,ti,kw OR telecare:ab,ti,kw OR ‘tele care’:ab,ti,kw OR telecommunicat*:ab,ti,kw OR ‘tele communicat*’:ab,ti,kw OR teleconference*:ab,ti,kw OR ‘tele conferenc*’:ab,ti,kw OR videoconferenc*:ab,ti,kw OR ‘video conferenc*’:ab,ti,kw OR computer*:ab,ti,kw OR electronic*:ab,ti,kw OR digital*:ab,ti,kw OR ehealth:ab,ti,kw OR ‘e health’:ab,ti,kw OR ‘e treat*’:ab,ti,kw OR ‘e therap*’:ab,ti,kw OR mhealth:ab,ti,kw OR ‘m health’:ab,ti,kw OR ‘internet based*’:ab,ti,kw OR ‘internet treat*’:ab,ti,kw OR ‘internet intervention*’:ab,ti,kw OR ‘internet counsel*’:ab,ti,kw OR ‘distance counsel*’:ab,ti,kw OR ‘web based*’:ab,ti,kw OR cybercounsel*:ab,ti,kw OR ‘cyber counsel*’:ab,ti,kw OR ‘online treat*’:ab,ti,kw OR ‘online therap*’:ab,ti,kw OR ‘online intervention*’:ab,ti,kw OR ‘online prevention*’:ab,ti,kw OR ‘online counsel*’:ab,ti,kw OR ‘text messag*’:ab,ti,kw OR textmessag*:ab,ti,kw OR SMS:ab,ti,kw OR texting*:ab,ti,kw OR ‘short message service*’:ab,ti,kw OR mobile*:ab,ti,kw OR smartphone*:ab,ti,kw OR ‘cell phone*’:ab,ti,kw OR cellphone*:ab,ti,kw OR ‘cellular phone*’:ab,ti,kw OR blended*:ab,ti,kw OR ‘software app*’:ab,ti,kw OR ‘handheld device*’:ab,ti,kw OR ‘hand held device*’:ab,ti,kw OR iPad*:ab,ti,kw OR iPhone*:ab,ti,kw OR email*:ab,ti,kw OR ‘e-mail*’:ab,ti,kw OR sensor*:ab,ti,kw OR wearable*:ab,ti,kw OR ‘social media*’:ab,ti,kw OR ‘social network*’:ab,ti,kw OR ‘e counsel*’:ab,ti,kw OR ecounsel*:ab,ti,kw OR palmtop*:ab,ti,kw OR telephone*:ab,ti,kw OR WhatsApp:ab,ti,kw OR Twitter:ab,ti,kw OR Facebook:ab,ti,kw OR Instagram:ab,ti,kw OR forum:ab,ti,kw OR chat*:ab,ti,kw OR ‘virtual reality*’:ab,ti,kw OR avatar*:ab,ti,kw OR ‘Conversational agent*’:ab,ti,kw OR ‘virtual coach’:ab,ti,kw OR ‘virtual agent*’:ab,ti,kw OR ‘embodied agent*’:ab,ti,kw OR ‘relational agent*’:ab,ti,kw OR ‘interactive agent*’:ab,ti,kw OR ‘virtual character*’:ab,ti,kw OR ‘virtual human*’:ab,ti,kw OR ‘virtual assistant*’:ab,ti,kw OR VR:ab,ti,kw OR ‘serious game*’:ab,ti,kw OR ‘serious gaming’:ab,ti,kw OR gamification:ab,ti,kw

**# SR + MA filter**

'meta analysis'/exp OR 'meta analysis (topic)'/exp OR 'systematic review'/exp OR 'systematic review (topic)'/exp OR metaanaly*:ab,ti,kw OR meta-analy*:ab,ti,kw or metanaly*:ab,ti,kw OR 'cochrane database of systematic reviews'/jt OR prisma:ab,ti,kw OR ‘preferred reporting items’:ab,ti,kw OR prospero:ab,ti,kw OR ‘systematic review’:ab,ti,kw OR ‘umbrella review’:ab,ti,kw OR ‘structured literature review’:ab,ti,kw OR ‘systematic qualitative review’:ab,ti,kw OR ‘systematic quantitative review’:ab,ti,kw OR ‘systematized review’:ab,ti,kw OR ‘systematised review’:ab,ti,kw OR ‘systemic review’:ab,ti,kw OR ‘systematic literature review’:ab,ti,kw OR ‘systematic integrative literature review’:ab,ti,kw OR ‘systematically review’:ab,ti,kw OR ‘scoping literature review’:ab,ti,kw OR ‘scoping review’:ab,ti,kw OR ‘systematic critical review’:ab,ti,kw OR ‘systematic integrative review’:ab,ti,kw OR ‘systematic evidence review’:ab,ti,kw OR ‘systematic integrative literature review’:ab,ti,kw OR ‘systematic mixed studies review’:ab,ti,kw OR ‘systematized literature review’:ab,ti,kw OR ‘systematic overview’:ab,ti,kw OR ‘Systematic narrative review’:ab,ti,kw OR ‘narrative review’:ab,ti,kw OR metasynthes*:ab,ti,kw OR meta-synthes*:ab,ti,kw OR 'clinical trial'/de OR 'double blind procedure'/de OR 'phase 1 clinical trial topic'/de OR 'phase 2 clinical trial topic'/de OR 'phase 3 clinical trial'/de OR 'phase 3 clinical trial topic'/de OR 'randomized controlled trial'/de OR 'single blind procedure'/de OR random:ab,ti,kw OR randomly:ab,ti,kw OR randomised:ab,ti,kw OR randomized:ab,ti,kw OR randomising:ab,ti,kw OR randomizing:ab,ti,kw OR RCT:ab,ti,kw OR ‘clinical trial*’:ab,ti,kw OR ‘controlled trial*’:ab,ti,kw

**# Publicationtype**

('article'/it OR 'article in press'/it OR 'conference paper'/it OR 'review'/it OR 'short survey'/it)

No congress abstracts – preprints included

Search strings for PsycINFO:

**# Depression**

DE "Major Depression" OR DE "Anaclitic Depression" OR DE "Dysthymic Disorder" OR DE "Endogenous Depression" OR DE "Late Life Depression" OR DE "Postpartum Depression" OR DE "Reactive Depression" OR DE "Recurrent Depression" OR DE "Treatment Resistant Depression" OR DE "Depression (Emotion)" OR TI(“depress*” OR “dysthymi*” OR “mood disorder*” OR “affective disorder*” OR “dysphoric disorder*”) OR AB(“depress*” OR “dysthymi*” OR “mood disorder*” OR “affective disorder*” OR “dysphoric disorder*”) OR KW(“depress*” OR “dysthymi*” OR “mood disorder*” OR “affective disorder*” OR “dysphoric disorder*”)

**# Psychotherapy**

DE "Psychotherapy" OR DE "Adlerian Psychotherapy" OR DE "Adolescent Psychotherapy" OR DE "Affirmative Therapy" OR DE "Analytical Psychotherapy" OR DE "Autogenic Training" OR DE "Brief Psychotherapy" OR DE "Brief Relational Therapy" OR DE "Child Psychotherapy" OR DE "Client Centered Therapy" OR DE "Couples Therapy" OR DE "Eclectic Psychotherapy" OR DE "Emotion Focused Therapy" OR DE "Existential Therapy" OR DE "Experiential Psychotherapy" OR DE "Expressive Psychotherapy" OR DE "Eye Movement Desensitization Therapy" OR DE "Feminist Therapy" OR DE "Gestalt Therapy" OR DE "Empty Chair Technique" OR DE "Group Psychotherapy" OR DE "Encounter Group Therapy" OR DE "Therapeutic Community" OR DE "Guided Imagery" OR DE "Humanistic Psychotherapy" OR DE "Client Centered Therapy" OR DE "Individual Psychotherapy" OR DE "Insight Therapy" OR DE "Integrative Psychotherapy" OR DE "Schema Therapy" OR DE "Interpersonal Psychotherapy" OR DE "Logotherapy" OR DE "Narrative Therapy" OR DE "Network Therapy" OR DE "Persuasion Therapy" OR DE "Primal Therapy" OR DE "Psychodrama" OR DE "Psychodynamic Psychotherapy" OR DE "Psychotherapeutic Counseling" OR DE "Family Therapy" OR DE "Psychotherapeutic Techniques" OR DE "Active Listening" OR DE "Animal Assisted Therapy" OR DE "Autogenic Training" OR DE "Brief Relational Therapy" OR DE "Centering" OR DE "Cotherapy" OR DE "Dream Analysis" OR DE "Empty Chair Technique" OR DE "Ericksonian Psychotherapy" OR DE "Free Association" OR DE "Guided Imagery" OR DE "Life Review" OR DE "Mirroring" OR DE "Morita Therapy" OR DE "Motivational Interviewing" OR DE "Mutual Storytelling Technique" OR DE "Network Therapy" OR DE "Paradoxical Techniques" OR DE "Psychodrama" OR DE "Rational Emotive Behavior Therapy" OR DE "Reality Therapy" OR DE "Relationship Therapy" OR DE "Solution Focused Therapy" OR DE "Strategic Therapy" OR DE "Strategic Family Therapy" OR DE "Supportive Psychotherapy" OR DE "Transactional Analysis" OR DE "Cognitive Therapy" OR TI(psychotherap* OR cbt OR counselling OR counseling OR “Eye Movement Desensitization Reprocessing” OR “Eye Movement Desensitization and Reprocessing” OR “Eye Movement Desensitisation Reprocessing” OR “Eye Movement Desensitisation and Reprocessing” OR EMDR OR “Bibliotherap*” OR mindfulness OR “Autogenic Training” OR Logotherap* OR "cognitive restructuring" OR "self-control training*" OR "assertiveness training" OR ((therap* OR treatment* OR intervention*) AND (“brief psychodynamic” OR “short psychodynamic” OR "problem-solving" OR "compassion-focused" OR "compassion-focussed" OR “compassion-based” OR constructivist* OR metacognitive OR “meta-cognitive” OR "solution-focused" OR "solution-focussed" OR "self-control” OR psychosocial OR “peer support“ OR “task-shifted” OR Relaxation OR “dialectical behavior” OR “emotion-focused” OR narrative OR “person-centred” OR “person-centered” OR “Narrative” OR “meaning-centered” OR “humanistic” OR “client-centered” OR “meaning-centred” OR “client-centred” OR “Rogerian” OR “Nondirective” OR “Non-directive” OR “Supportive” OR “Life review” OR "acceptance and commitment" OR (“schema” AND brief) OR (“gestalt” AND brief))) OR "behavior therap*" OR "behaviors therap*" OR "behavioral therap*" OR "behaviour therap*" OR "behaviours therap*" OR "behavioural therap*" OR "cognition therap*" OR “cognitive therap*” OR "behavior treatment*" OR "behaviors treatment*" OR "behavioral treatment*" OR "behaviour treatment*" OR "behaviours treatment*" OR "behavioural treatment*" OR "cognition treatment*" OR “cognitive treatment*” OR "behavior intervention*" OR "behaviors intervention*" OR "behavioral intervention*" OR "behaviour intervention*" OR "behaviours intervention*" OR "behavioural intervention*" OR "cognition intervention*" OR “cognitive intervention*” OR "behavior activation*" OR "behaviors activation*" OR "behavioral activation*" OR "behaviour activation*" OR "behaviours activation*" OR "behavioural activation*" OR exposure) OR AB((psychotherap* OR cbt OR counselling OR counseling OR “Eye Movement Desensitization Reprocessing” OR “Eye Movement Desensitization and Reprocessing” OR “Eye Movement Desensitisation Reprocessing” OR “Eye Movement Desensitisation and Reprocessing” OR EMDR OR “Bibliotherap*” OR mindfulness OR “Autogenic Training” OR Logotherap* OR "cognitive restructuring" OR "self-control training*" OR "assertiveness training" OR ((therap* OR treatment* OR intervention*) AND (“brief psychodynamic” OR “short psychodynamic” OR "problem-solving" OR "compassion-focused" OR "compassion-focussed" OR “compassion-based” OR constructivist* OR metacognitive OR “meta-cognitive” OR "solution-focused" OR "solution-focussed" OR "self-control” OR psychosocial OR “peer support“ OR “task-shifted” OR Relaxation OR “dialectical behavior” OR “emotion-focused” OR narrative OR “person-centred” OR “person-centered” OR “Narrative” OR “meaning-centered” OR “humanistic” OR “client-centered” OR “meaning-centred” OR “client-centred” OR “Rogerian” OR “Nondirective” OR “Non-directive” OR “Supportive” OR “Life review” OR "acceptance and commitment" OR (“schema” AND brief) OR (“gestalt” AND brief))) OR "behavior therap*" OR "behaviors therap*" OR "behavioral therap*" OR "behaviour therap*" OR "behaviours therap*" OR "behavioural therap*" OR "cognition therap*" OR “cognitive therap*” OR "behavior treatment*" OR "behaviors treatment*" OR "behavioral treatment*" OR "behaviour treatment*" OR "behaviours treatment*" OR "behavioural treatment*" OR "cognition treatment*" OR “cognitive treatment*” OR "behavior intervention*" OR "behaviors intervention*" OR "behavioral intervention*" OR "behaviour intervention*" OR "behaviours intervention*" OR "behavioural intervention*" OR "cognition intervention*" OR “cognitive intervention*” OR "behavior activation*" OR "behaviors activation*" OR "behavioral activation*" OR "behaviour activation*" OR "behaviours activation*" OR "behavioural activation*" OR exposure) OR KW(psychotherap* OR cbt OR counselling OR counseling OR “Eye Movement Desensitization Reprocessing” OR “Eye Movement Desensitization and Reprocessing” OR “Eye Movement Desensitisation Reprocessing” OR “Eye Movement Desensitisation and Reprocessing” OR EMDR OR “Bibliotherap*” OR mindfulness OR “Autogenic Training” OR Logotherap* OR "cognitive restructuring" OR "self-control training*" OR "assertiveness training" OR ((therap* OR treatment* OR intervention*) AND (“brief psychodynamic” OR “short psychodynamic” OR "problem-solving" OR "compassion-focused" OR "compassion-focussed" OR “compassion-based” OR constructivist* OR metacognitive OR “meta-cognitive” OR "solution-focused" OR "solution-focussed" OR "self-control” OR psychosocial OR “peer support“ OR “task-shifted” OR Relaxation OR “dialectical behavior” OR “emotion-focused” OR narrative OR “person-centred” OR “person-centered” OR “Narrative” OR “meaning-centered” OR “humanistic” OR “client-centered” OR “meaning-centred” OR “client-centred” OR “Rogerian” OR “Nondirective” OR “Non-directive” OR “Supportive” OR “Life review” OR "acceptance and commitment" OR (“schema” AND brief) OR (“gestalt” AND brief))) OR "behavior therap*" OR "behaviors therap*" OR "behavioral therap*" OR "behaviour therap*" OR "behaviours therap*" OR "behavioural therap*" OR "cognition therap*" OR “cognitive therap*” OR "behavior treatment*" OR "behaviors treatment*" OR "behavioral treatment*" OR "behaviour treatment*" OR "behaviours treatment*" OR "behavioural treatment*" OR "cognition treatment*" OR “cognitive treatment*” OR "behavior intervention*" OR "behaviors intervention*" OR "behavioral intervention*" OR "behaviour intervention*" OR "behaviours intervention*" OR "behavioural intervention*" OR "cognition intervention*" OR “cognitive intervention*” OR "behavior activation*" OR "behaviors activation*" OR "behavioral activation*" OR "behaviour activation*" OR "behaviours activation*" OR "behavioural activation*" OR exposure)

**# Internet-based**

DE "Computer Assisted Therapy" OR DE "Online Therapy" OR DE "Teleconsultation" OR DE "Telepsychiatry" OR DE "Telepsychology" OR DE "Telerehabilitation" OR DE "Digital Interventions" OR DE "Telemedicine" OR DE "Videoconferencing" OR DE "Telecommunications Media" OR DE "Teleconferencing" OR DE "Smartphones" OR DE "Mobile Applications" OR DE "Mobile Devices" OR DE "Mobile Phones" OR DE "Tablet Computers" OR DE "Mobile Health" OR DE "Text Messaging" OR DE "Wearable Devices" OR DE "Virtual Reality" OR DE "Augmented Reality" OR TI(telehealth OR “tele-health” OR telepsychology OR “tele-psychology” OR telepsychiatry OR “tele-psychiatry” OR “tele-therap*” OR teletherap* OR “tele-medicine” OR telemedicine OR telecare OR “tele-care” OR telecommunicat* OR “tele-communicat*” OR teleconference* OR “tele-conferenc*” OR videoconferenc* OR “video-conferenc*” OR computer* OR electronic* OR digital* OR ehealth OR “e-health” OR “e-treat*” OR “e-therap*” OR mhealth OR “m-health” OR “internet-based*” OR “internet treat*” OR “internet intervention*” OR “internet counsel*” OR “distance counsel*” OR “web-based*” OR cybercounsel* OR “cyber-counsel*” OR “online treat*” OR “online therap*” OR “online intervention*” OR “online prevention*” OR “online counsel*” OR “text-messag*” OR textmessag* OR SMS OR texting* OR “short message service*” OR mobile* OR smartphone* OR “cell-phone*” OR cellphone* OR “cellular phone*” OR blended* OR “software app*” OR “handheld device*” OR “hand held device*” OR iPad* OR iPhone* OR email* OR “e-mail*” OR sensor* OR wearable* OR “social media*” OR “social network*” OR “e-counsel*” OR ecounsel* OR palmtop* OR telephone* OR WhatsApp OR Twitter OR Facebook OR Instagram OR forum OR chat* OR “virtual reality*” OR avatar* OR “Conversational agent*” OR “virtual coach” OR “virtual agent*” OR “embodied agent*” OR “relational agent*” OR “interactive agent*” OR “virtual character*” OR “virtual human*” OR “virtual assistant*” OR VR OR “serious game*” OR “serious gaming” OR gamification) OR AB(telehealth OR “tele-health” OR telepsychology OR “tele-psychology” OR telepsychiatry OR “tele-psychiatry” OR “tele-therap*” OR teletherap* OR “tele-medicine” OR telemedicine OR telecare OR “tele-care” OR telecommunicat* OR “tele-communicat*” OR teleconference* OR “tele-conferenc*” OR videoconferenc* OR “video-conferenc*” OR computer* OR electronic* OR digital* OR ehealth OR “e-health” OR “e-treat*” OR “e-therap*” OR mhealth OR “m-health” OR “internet-based*” OR “internet treat*” OR “internet intervention*” OR “internet counsel*” OR “distance counsel*” OR “web-based*” OR cybercounsel* OR “cyber-counsel*” OR “online treat*” OR “online therap*” OR “online intervention*” OR “online prevention*” OR “online counsel*” OR “text-messag*” OR textmessag* OR SMS OR texting* OR “short message service*” OR mobile* OR smartphone* OR “cell-phone*” OR cellphone* OR “cellular phone*” OR blended* OR “software app*” OR “handheld device*” OR “hand held device*” OR iPad* OR iPhone* OR email* OR “e-mail*” OR sensor* OR wearable* OR “social media*” OR “social network*” OR “e-counsel*” OR ecounsel* OR palmtop* OR telephone* OR WhatsApp OR Twitter OR Facebook OR Instagram OR forum OR chat* OR “virtual reality*” OR avatar* OR “Conversational agent*” OR “virtual coach” OR “virtual agent*” OR “embodied agent*” OR “relational agent*” OR “interactive agent*” OR “virtual character*” OR “virtual human*” OR “virtual assistant*” OR VR OR “serious game*” OR “serious gaming” OR gamification) OR KW(telehealth OR “tele-health” OR telepsychology OR “tele-psychology” OR telepsychiatry OR “tele-psychiatry” OR “tele-therap*” OR teletherap* OR “tele-medicine” OR telemedicine OR telecare OR “tele-care” OR telecommunicat* OR “tele-communicat*” OR teleconference* OR “tele-conferenc*” OR videoconferenc* OR “video-conferenc*” OR computer* OR electronic* OR digital* OR ehealth OR “e-health” OR “e-treat*” OR “e-therap*” OR mhealth OR “m-health” OR “internet-based*” OR “internet treat*” OR “internet intervention*” OR “internet counsel*” OR “distance counsel*” OR “web-based*” OR cybercounsel* OR “cyber-counsel*” OR “online treat*” OR “online therap*” OR “online intervention*” OR “online prevention*” OR “online counsel*” OR “text-messag*” OR textmessag* OR SMS OR texting* OR “short message service*” OR mobile* OR smartphone* OR “cell-phone*” OR cellphone* OR “cellular phone*” OR blended* OR “software app*” OR “handheld device*” OR “hand held device*” OR iPad* OR iPhone* OR email* OR “e-mail*” OR sensor* OR wearable* OR “social media*” OR “social network*” OR “e-counsel*” OR ecounsel* OR palmtop* OR telephone* OR WhatsApp OR Twitter OR Facebook OR Instagram OR forum OR chat* OR “virtual reality*” OR avatar* OR “Conversational agent*” OR “virtual coach” OR “virtual agent*” OR “embodied agent*” OR “relational agent*” OR “interactive agent*” OR “virtual character*” OR “virtual human*” OR “virtual assistant*” OR VR OR “serious game*” OR “serious gaming” OR gamification)

**# SR + MA filter**

TI(metaanaly* OR meta-analy* or metanaly* OR prisma OR “preferred reporting items” OR prospero OR “systematic review” OR “umbrella review” OR “structured literature review” OR “systematic qualitative review” OR “systematic quantitative review” OR “systematic search and review” OR “systematized review” OR “systematised review” OR “systemic review” OR “systematic literature review” OR “systematic integrative literature review” OR “systematically review” OR “scoping literature review” OR “scoping review” OR “systematic critical review” OR “systematic integrative review” OR “systematic evidence review” OR “systematic integrative literature review” OR “systematic mixed studies review” OR “systematized literature review” OR “systematic overview” OR “Systematic narrative review” OR “narrative review” OR metasynthes* OR meta-synthes* OR random OR randomly OR randomised OR randomized OR randomising OR randomizing OR RCT OR “clinical trial*” OR “controlled trial*”) OR AB(metaanaly* OR meta-analy* or metanaly* OR prisma OR “preferred reporting items” OR prospero OR “systematic review” OR “umbrella review” OR “structured literature review” OR “systematic qualitative review” OR “systematic quantitative review” OR “systematic search and review” OR “systematized review” OR “systematised review” OR “systemic review” OR “systematic literature review” OR “systematic integrative literature review” OR “systematically review” OR “scoping literature review” OR “scoping review” OR “systematic critical review” OR “systematic integrative review” OR “systematic evidence review” OR “systematic integrative literature review” OR “systematic mixed studies review” OR “systematized literature review” OR “systematic overview” OR “Systematic narrative review” OR “narrative review” OR metasynthes* OR meta-synthes* OR random OR randomly OR randomised OR randomized OR randomising OR randomizing OR RCT OR “clinical trial*” OR “controlled trial*”) OR KW(metaanaly* OR meta-analy* or metanaly* OR prisma OR “preferred reporting items” OR prospero OR “systematic review” OR “umbrella review” OR “structured literature review” OR “systematic qualitative review” OR “systematic quantitative review” OR “systematic search and review” OR “systematized review” OR “systematised review” OR “systemic review” OR “systematic literature review” OR “systematic integrative literature review” OR “systematically review” OR “scoping literature review” OR “scoping review” OR “systematic critical review” OR “systematic integrative review” OR “systematic evidence review” OR “systematic integrative literature review” OR “systematic mixed studies review” OR “systematized literature review” OR “systematic overview” OR “Systematic narrative review” OR “narrative review” OR metasynthes* OR random OR randomly OR randomised OR randomized OR randomising OR randomizing OR RCT OR “clinical trial*” OR “controlled trial*”)

Methodology Filter:

Narrow by Methodology: - meta analysis

Narrow by Methodology: - systematic review

Narrow by Methodology - Clinical trial

**# Publication type**

Academic journals
